# Overexpression of chloride channel-3 (ClC-3) is associated with human cervical carcinoma development and prognosis

**DOI:** 10.1186/s12935-018-0721-x

**Published:** 2019-01-08

**Authors:** Yu-tao Guan, Yong Xie, Hui Zhou, Hai-yan Shi, Yu-yuan Zhu, Xiao-lu Zhang, Yi Luan, Xi-ming Shen, Yang-ping Chen, Li-jiang Xu, Zhong-qiu Lin, Gang Wang

**Affiliations:** 1 Department of Obstetrics and Gynaecology, Sun Yat-sen Memorial Hospital of Sun Yat-Sen University, Guangzhou, China; 2 Department of Obstetrics and Gynaecology, The First People’s Hospital of Foshan, Foshan, China; 3 Department of Pathology, The First People’s Hospital of Foshan, Foshan, China; 4 Department of Pathology, Sun Yat-Sen Memorial Hospital of Sun Yat-Sen University, Guangzhou, China

**Keywords:** Chloride channel-3 (ClC-3), Cervical carcinoma, Development, Prognosis

## Abstract

**Background:**

Cervical carcinoma is a major gynecological cancer and causes cancer-related deaths in worldwide, the latent pathogenesis and progress of cervical cancer is still under research. ClC-3 may be an important promoter for aggressive metastasis of malignant tumors. In this research, we explore the ClC-3 expression in cervical carcinoma and its underlying clinical significance, trying to illuminate ClC-3 probable function in the neoplasm malignant behavior, development and prognosis.

**Methods:**

Paraffin-embedded cervical (n = 168) and lymph node (n = 100) tissue specimens were analysed by immunohistochemistry. Fresh human cervical tissue specimens (n = 165) and four human cervical cell lines were tested for ClC-3 mRNA and protein expression levels by quantitative real-time PCR and western blotting. The relationship between the expression levels of ClC-3, the pathological characteristics of the carcinoma, and the clinical prognosis were statistically analysed.

**Results:**

In normal and precancerous (LSIL, HSIL) cervical tissues as well as cervical carcinoma tissues, both ClC-3 mRNA and protein expression levels increased significantly (*p *< 0.05). The expression level of ClC-3 was closely-related to the histological differentiation (p = 0.029), tumour staging (*p *= 0.016), tumour size (*p *= 0.039), vascular invasion (*p *= 0.045), interstitial infiltration depth (*p *= 0.012), lymphatic metastasis (*p *= 0.036), and HPV infection (*p *= 0.022). In an in vitro experiment, ClC-3 mRNA and protein were found to be overexpressed both in the HeLa and SiHa cell lines, but low expression levels were detected in the C-33A and H8 cell lines (*p *< 0.05). Furthermore, the high expression levels of ClC-3 was significantly correlated to poor survival in cervical carcinoma patients (Log-rank test, *p *= 0.046).

**Conclusions:**

These data suggest that overexpression of ClC-3 is closely associated with human cervical carcinoma progression and poor prognosis; this suggests that ClC-3 may function as a patent tumour biomarker and a latent therapeutic target for cervical carcinoma patients.

## Background

Cervical carcinoma is a major gynaecological cancer that causes thousands of cancer-related deaths in women worldwide; most cervical carcinoma diagnoses occur in developing countries [1]. The most common type of cervical cancer is squamous cell carcinoma (SCC), which develops from low-squamous intraepithelial lesions (LSIL) and high-squamous intraepithelial lesions (HSIL). The main risk factor for cervical carcinoma is papillomavirus (HPV) infection [2]. More than 80% of women have been infected with HPV, but only a small proportion of women develop cervical cancer. This suggests that some other factors may take part in the pathogenesis of cervical cancer. Therefore, the underlying pathogenesis and progress of cervical cancer is still being investigated.

Studies have found that membrane ion channels play a significant part in the progress and metastasis of malignant tumours [3]. Chloride channels have been documented to promote tumour cell invasion and the formation of brain metastasis in primary brain tumours and glioma [4]; chloride channel-3 (ClC-3) has been proven to take part in cell migration and invasion [5, 6], indicating ClC-3 can be a crucial promoter of metastasis.

ClC-3 is a member of the voltage-gated Cl− channel superfamily [7] and is implicated in the regulation of malignant tumour cell behaviour such as proliferation, migration, invasion, and apoptosis [8–10]. Previously, our studies found that ClC-3 plays a key role in ectopic endometrial cell migration and invasion [5]. Recent studies show that ClC-3 plays an active and vital role in accelerating neoplasm metastasis and may be a prognostic biomarker of tumour dissemination [11]. Oestrogen can activate expression and promote translation of the ClC-3 gene [12], and be responsible for promoting cancer growth [13]. ClC-3 abnormal expression and dysfunction may result in various pathological conditions [14]. Notably, several studies have recently shown that changes in expression of the ClC-3 gene may augment the risk of developing a variety of cancers, including endometrial carcinoma [15], nasopharyngeal carcinoma [16], breast cancer [13] and glioma [17]. ClC-3 has been shown to actively participate in various molecular signal pathways that facilitate the aggressiveness and metastasis of malignant tumours [9]. These data show that ClC-3 may play a crucial role in the occurrence and development of different kinds of cancers.

In this study, we investigate ClC-3 expression in cervical carcinoma and its underlying clinical significance, and we attempt to elucidate the probable function of ClC-3 in malignant neoplasm behaviour, development and prognosis.

## Materials and methods

### Ethical authorization

This research conforms to and acts in accordance with the Enhancing the QUAlity and Transparency Of health Research (EQUATOR) guidelines (http://www.equatornetwork.org/). All fresh human tissue specimens were collected by informed consent following the requirements of the Research Ethics Committee of the Foshan First People’s Hospital from February 2017 to December 2017.

### Human tissue collection

The following cervical tissue specimens were collected and paraffin-embedded: normal cervical tissues (N, n = 30), LSIL (n = 35), HSIL (n = 43), SCC (n = 60), lymph node metastasis positive (LP, n = 50) and lymph node metastasis negative (LN, n = 50); the tissues were then evaluated via immunohistochemistry (IHC) by the Pathology Department at the Sun Yat-Sen Memorial Hospital of Sun Yat-Sen University in 2012.

The fresh cervical tissue cohort included cervical tissue (N, n = 45) from Han Chinese patients who accepted laparoscopic surgery for myoma of the uterus, cervical squamous cell carcinoma (SCC, n = 60) and the relevant paracancerous normal cervical tissues (PN, n = 60) from patients who accepted laparoscopic surgery for cervical cancer confirmed by pathological examination. Specimens were collected from the Department of Gynaecology and Obstetrics (The First People’s Hospital of Foshan, Sun Yat-Sen University) during 2017 for protein and mRNA analyses. The relevant paracancerous normal cervical tissues were acquired from 3 cm over the margin of cervical carcinoma tissues. According to the International Federation of Gynaecological and Obstetrics (FIGO) classification in 2009, the carcinoma patients were in stages from IB to IIA. The patients did not receive any neoadjuvant chemotherapy or radiotherapy before the radical operation.

### Cell culture

HeLa, SiHa, C-33A, H8 and HaCaT cell lines were purchased from GuangZhou Jennio Biotech Co., Ltd. and maintained in cell culture. The HeLa cell line was cultured from cervical adenocarcinoma infected by HPV-18, and the SiHa cell line was cultured from cervical squamous cell carcinoma infected by HPV-16; the C-33A cell line was cultured from HPV-negative cervical cancer, and H8 was cultured from normal cervical squamous epithelium as normal controls. The HaCaT cell line was derived from a normal keratinocyte. All cells were cultured with DMEM (GIBCO) containing 10% foetal calf serum (HyClone, USA) and double antibiotics (100 Units/ml penicillin and 100 μg/ml streptomycin) (GIBCO). The cells were incubated in a humidified atmosphere with 5% CO2 at 37 °C. The cell lines were authenticated.

### HPV test and genotyping

HPV genomic DNA from the paraffin-embedded cervical carcinoma specimens (n = 65) was gathered and tested with the TaKaRa DEXPAT kit (TaKaRa). The HPV DNA and genotypes were tested in the Hospital Clinical laboratory with the HPV GenoArray kit (HybriBio) following the manufacturer’s instructions. The assay was able to identify 2 high-risk types (16, 18) and other HPV genotypes.

### Quantitative real-time PCR

The mRNA expression levels from fresh cervical tissues and cultured cells were detected with the real-time RT-PCR (CFX96 real-time system, Bio-Rad, USA) by using the SYBR Green fluorescence signal test kit according to the manufacturer’s instructions. The real-time RT-PCR quantified the mRNA expression levels and was carried out in triplicate for each sample. The cycling conditions were set at 95 °C for 5 min, followed by 40 cycles at 95 °C for 10 s, finally, 60 °C for 5 s and 72 °C for 15 s. The sequences of the primer pairs for human ClC-3 were as follows: forward primer 5′-TTGCCTACTATCACCACGAC-3′ and reverse primer 5′-GCATCTCCAACCCATTTACT-3′. The human glyceraldehyde-3-phosphate dehydrogenase (GAPDH) gene was amplified as an internal reference using the following primers: 5′-GGTGGTCTCCTCTGACTTCAACA-3′ (forward) and 5′-GTTGCTGTAGCCAAATTCGTTGT-3′ (reverse). The 2^−ΔΔCt^ method measured the relative gene expression levels (folds), and all the tests were performed at least three times [5].

### Immunohistochemistry assay

Routine deparaffinization and rehydration were performed for the paraffin-embedded cervical tissue samples. Antigen retrieval was accomplished in 10 mM sodium citrate buffer, pH 6.0, for 10 min at 92–98 °C; the samples were then treated with 0.3% H_2_O_2_ for 15 min. Normal goat serum incubation was performed for 20 min to eliminate nonspecific binding, and after washing with PBS, the samples were incubated in the mouse anti-ClC-3 antibody (1:500 dilution, Abcam, Cambridge, MA, USA) at 4 °C overnight. The sections were washed with PBS and incubated in biotinylated secondary antibody for 60 min (1:800 dilution, Beyotime Biotechnology Inc.). The sections were then processed in ABC solution for 30 min at 37 °C prior to treatment with DAB (3,3′-diaminobenzidine) for 5 min. Counterstaining was performed with Harris haematoxylin. Positive staining was visualized as brown staining in the cell membrane or the cytoplasm.

The slides were imaged in an inverted microscope. For each slide, images of five random fields were acquired; using the Image-Pro Plus 6.0 image analysis software (Media Cybernetics, Rockville, MD) the mean immunostaining density was measured as described before [5].

For immunochemistry of the cultured cells, cultured cells were incubated in 24-well plates for 24 h and fixed with paraformaldehyde (4%) and sucrose (0.12 M) in PBS. The cells were permeabilized with Triton X-100 (0.5% in PBS), blocked with 10% normal sheep serum in PBS for 20 min and treated with the anti-ClC-3 antibody and the biotinylated secondary antibody following the similar procedures as described above.

### Western blot assay

The total protein levels of the fresh cervical tissue specimens (N = 45, SCC = 60, PN = 60) and cultured cells were evaluated by using the BCA assay kit (SinoBio Biotech) to measure protein concentration which was performed following the manufacturer’s instructions. Equal amounts of protein were analysed by electrophoresis in 8% SDS-PAGE gels and transferred to polyvinylidene fluoride membranes (Millipore, Bedford, MA). The membranes were blocked using 5% skim milk for 2 h at room temperature. Subsequently, the membranes were incubated with the mouse monoclonal anti-ClC-3 antibody (diluted 1:1000, Abcam) or the mouse anti-ß-actin antibody (diluted 1:1000, Beyotime Biotechnology Inc.) overnight at 4 °C. The membranes were then incubated in the HRP-conjugated secondary antibody for 2 h at room temperature. The proteins were measured by using the ECL system (CWBIOTECH, China) with the ChemiDoc XRS system (Bio-Rad, Philadelphia, USA).

### Statistics

Statistical analyses were carried out by the SPSS 16.0 software (SPSS Inc., Chicago). The data were presented as the mean ± standard deviation (SD). The ClC-3 expression status of the clinicopathological samples was determined by using the Pearson’s *χ*^2^ test or the Fisher’s exact test. The analysis of variance and Student’s t-test were used to analyse the significant differences in the ClC-3 expression levels between the groups. The univariate cumulative survival was assessed by the Kaplan–Meier method and the log-rank test. Differences were considered significant at *p *< 0.05.

## Results

### Increased ClC-3 mRNA expression in cervical cancer tissue

**Fig. 1:**
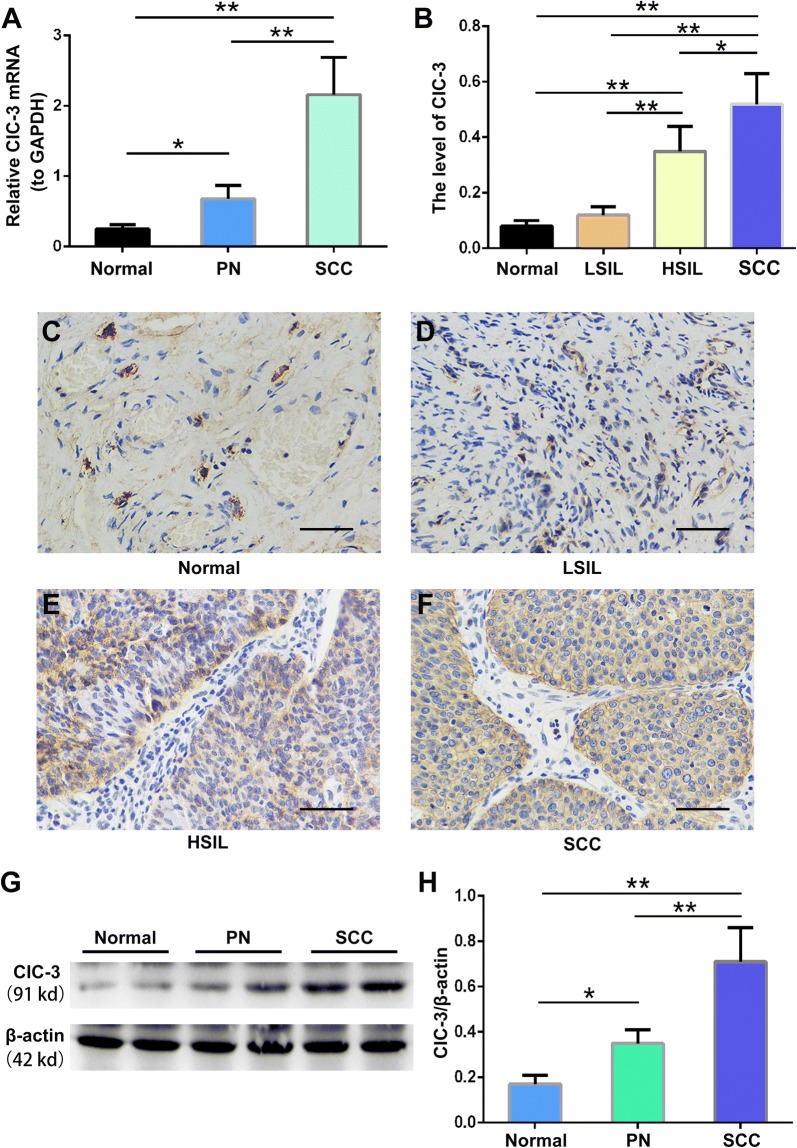
Increased expression levels of the ClC-3 mRNA and protein in paracancerous and carcinoma tissues. **A** ClC-3 mRNA expression levels were detected by quantitative real time RT-PCR in the control, non-cervical cancer, cervical samples (normal), the corresponding paracancerous normal tissues (PN) and the matched cancer samples (SCC) from 165 patients with cervical cancer. **B** Densitometric analysis of the ClC-3 protein expression levels in normal, low-squamous intraepithelial lesions (LSIL), high-squamous intraepithelial lesions (HSIL) and squamous cell carcinoma (SCC) tissues by immunohistochemistry. **C**–**F** Images of representative tissues showing the ClC-3 immunohistochemical staining. The main and inserted images were taken by a × 40 objective lens. Scale bars, 50 μm. **G** The representative western blot analysis of the ClC3 and β-actin proteins from normal, PN and SCC tissues. **H** Densitometric analysis of ClC3 protein levels in different tissues as detected by western blotting. The data in **A**, **B** and **E** are shown as the mean ± SD. *p < 0.05, **p < 0.01

The ClC-3 mRNA expression levels were measured in fresh tissue specimens (n = 165) from control (normal, non-cervical cancer) cervical specimens, the corresponding paracancerous normal tissues and the matched cancer specimens from the patients with cervical cancer. As displayed in Fig. 1A, the expression of ClC-3 mRNA gradually increased from normal to paracancerous to carcinoma tissues (N: 0.25 ± 0.0.06, PN: 0.68 ± 0.19, and SCC: 2.16 ± 0.53). There were significant differences between the normal tissues and paracancerous tissues (p < 0.05), and the normal and cancerous tissues (p < 0.01). Furthermore, we found the ClC-3 mRNA expression levels in the cervical cancer tissues were significantly larger than that in the corresponding paracancerous normal tissues (p < 0.01). From the 60 patients with cervical cancer, 81.7% of cancer specimens (49/60) presented an elevated ClC-3 expression level (compared with the paracancerous specimens, 2.45-fold difference, p < 0.01), and 18.3% of cancer specimens (11/60) did not express an elevated level of ClC-3 mRNA. These results suggest the expression level of ClC-3 mRNA is gradually upregulated in paracancerous and cancerous stages and is strongly associated with the evolution of cervical cancer.

### Increased ClC-3 protein expression from normal cervical tissue to cervical cancer tissue

To further confirm whether the ClC-3 protein expression levels change, we identified the ClC-3 expression in cervical tissues at the protein level by immunohistochemistry. The results collected from the paraffin-embedded samples are displayed in Fig. 1B–F. ClC-3 protein was mainly expressed in the cervix squamous epithelial cell cytoplasmic area. While only 10% of normal tissues expressed the ClC-3 protein, the protein levels were the highest (85%) in cervical carcinoma tissues (Table 1a). Importantly, positive ClC-3 protein expression was increased gradually from normal (10%) to precancerous (LSIL: 28.6% and HSIL: 65.1%) to carcinoma tissues (85%) suggesting ClC-3 protein expression is closely associated with the progression of cervical carcinoma (p < 0.001). Simultaneously, ClC-3 protein expression in the cervical carcinoma tissues and the relevant paracancerous normal tissues was detected (Fig. 1G, H). We detected that the ClC-3 protein levels in the normal tissues were very low and very high in the cervical cancer tissues. The positive results represented infiltration of cancer cells. ClC-3 protein expression in the corresponding paracancerous normal tissues was significantly weaker than that in the cervical carcinoma tissues (85% vs. 48.3%, p < 0.001) (Table 1b). These data correspond with the expression level changes of ClC-3 mRNA indicating that ClC-3 gene expression level is closely related to the development of cervical carcinoma.

**Table 1:** Expression of ClC-3 protein in cervical tissue

Group	Number	Number of ClC-3(+) cases (%)^a^	p^b^
a. CIC-3 protein expression in normal, precancerous and cervical cancer			
Normal	30	3 (10.0)	< 0.001
LSIL	35	10 (28.6)	
HSIL	43	28 (65.1)	
Cancer	60	51 (85.0)	
Total	168	92 (54.8)	
b. CIC-3 protein expression in the cervical cancer and the corresponding paracancerous normal tissues			
The cervical cancer tissues	60	51 (85.0)	< 0.001
The corresponding paracancerous normal tissues	60	29 (48.3)	
Total	120	80 (66.7)	

^a^ClC3(+) denotes overexpression of ClC3 protein

^b^By Pearson’s χ^2^ test or Fisher’s exact test

### Increased ClC-3 protein expression in the cancer positive lymph node

**Fig. 2:**
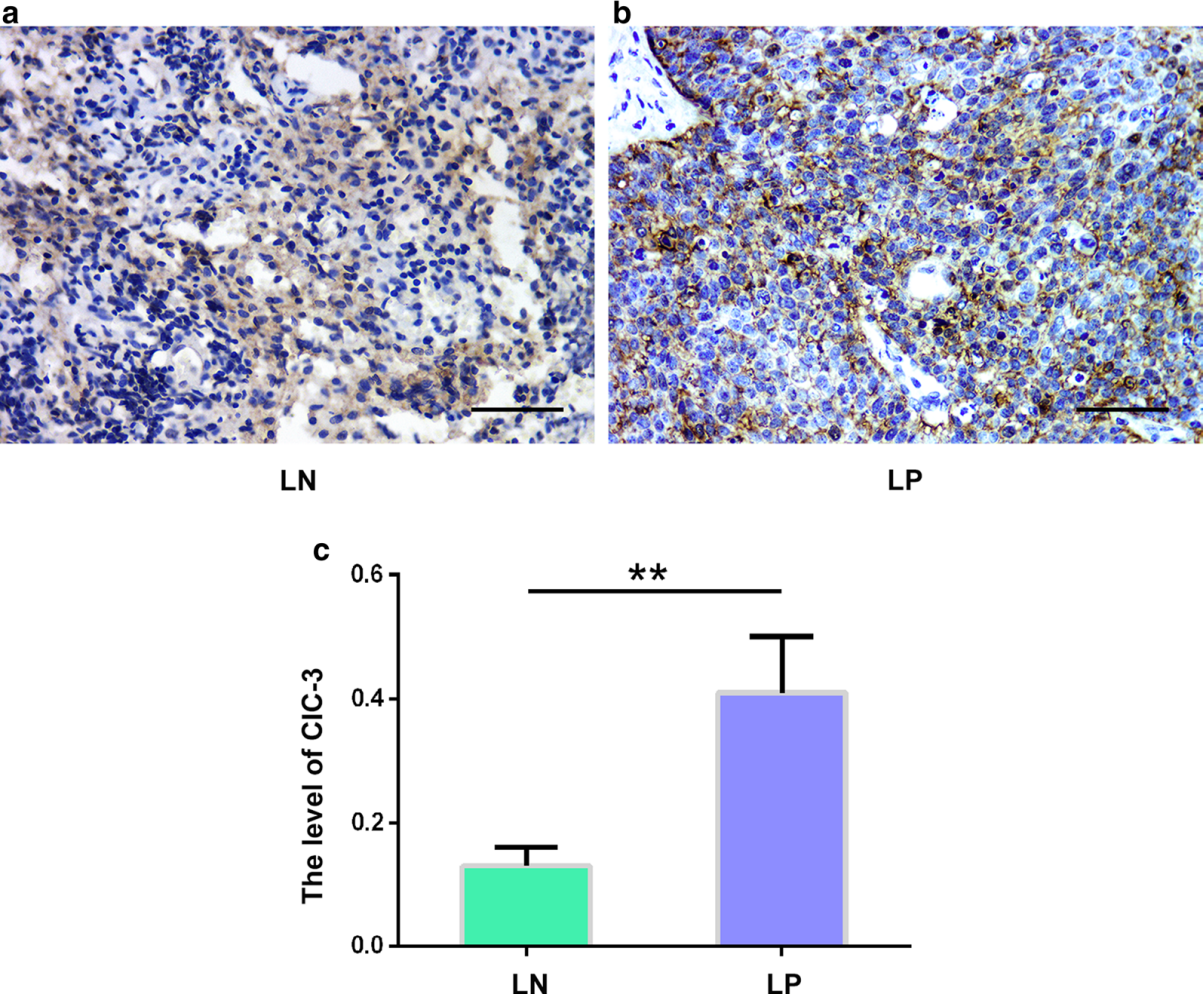
Increased expression levels of the ClC-3 protein in metastasis positive lymph nodes. **a**, **b** Images from lymph node metastasis positive (LP) and lymph node metastasis negative (LN) tissues showing the ClC-3 immunohistochemical staining. The main and inserted images were taken by using a × 40 objective lens. Scale bars, 50 μm. **c** Densitometric analysis of the ClC3 protein levels of LP and LN tissues detected by the immunohistochemistry. The data in **b** are shown as the mean ± SD. **p < 0.01

Our previous study found that ClC-3 overexpression facilitates cell migration and invasion [5]. It indicated that high ClC-3 expression levels may be closely related to cancer’s ability to metastasize. To verify this assumption, we identified the expression level of ClC-3 at the protein level using IHC in paraffin-embedded specimens derived from cervical cancer patients that were either lymph node metastasis positive (LP, n = 50) and lymph node metastasis negative (LN, n = 50). The data acquired from these paraffin-embedded specimens are displayed in Fig. 2, and we found that the ClC-3 protein level in the lymph nodes of metastatic cancer tissues was significantly higher than that in lymph node tissues without metastatic cancer (p < 0.05). In the 50 lymph node metastasis positive tissues, 86.0% of the cancer samples (43/50) showed increased expression level of ClC-3 protein (compared with the lymph node metastasis negative samples, 2.39-fold difference, p < 0.01), and 14.0% of the lymph node metastasis positive samples (7/50) did not show an elevated ClC-3 protein expression. These results suggested the expression level of the ClC-3 protein is closely related to the progression of cervical carcinoma.

### The relationship between the expression level of ClC-3 and clinical pathological characteristics

The above results raised the question of whether ClC-3 is related to the clinical features of cervical cancer. The relationship between the expression level of ClC-3 protein and the clinical pathological characteristics was studied in 60 cervical cancer patients with an average of 45.44 ± 11.3 years (Table 2). The data showed that the ClC-3 expression level was related to the tumour staging (*p *= 0.016), histological differentiation (*p *= 0.029), tumour size (*p *= 0.039), vascular invasion (*p *= 0.045), interstitial infiltration depth (*p *= 0.012), lymphatic metastasis (*p *= 0.036), and HPV infection (*p *= 0.022). The ClC-3 positive rate gradually increased from stage I to stage II (68.2% and 94.7%, respectively). However, the specimens in stage III and stage IV were not obtained to distinctly specify the function of ClC-3 expression in advanced cervical carcinoma, which may deviate the ClC-3 positive detection rate. Therefore, more samples are needed to elucidate this problem. Since tumour stage, histological grades, tumour size, vascular invasion, interstitial invasive depth, and lymphatic metastasis are considered key factors for carcinoma migration, invasion and metastasis, the results show that ClC-3 protein expression level is closely related to the malignancy degree of cervical carcinoma. In addition, the infection rate of HPV was 89.1% in patients with cervical carcinoma. HPV 16 was the most common in single and multiple HPV infections. The rate of ClC-3 expression in the HPV negative specimens was significantly lower than that in the HPV positive specimens (40.0% vs. 89.1%, *p *= 0.022). These results indicate the expression of ClC-3 protein was closely related to HPV infection in cervical cancer.

**Table 2:** Cervical cancer patient characteristics and ClC-3 expression

Characteristic	Total (60)	No. of CIC-3(+) cases (%)^a^	p^b^
Age (years)			
Mean (SD)	45.44 ± 11.3		
Stage			
I	22	15 (68.2%)	0.016
II	38	36 (94.7%)	
Histological grades			
HDSCC^c^	19	13 (68.4%)	0.029
MDSCC^c^	25	22 (88.0%)	
PDSCC^c^	16	16 (100.0%)	
Tumor size (cm)			
D ≤ 4	31	23 (74.2%)	0.039
D > 4	29	28 (96.6%)	
Vascular invasion			
Yes	21	21 (100.0%)	0.045
No	39	30 (76.9%)	
Invasive interstitial depth			
< 1/2	21	14 (66.7%)	0.012
≥ 1/2	28	26 (92.9%)	
Whole layer	11	11 (100.0%)	
Lymph node metastasis			
Yes	41	38 (92.7%)	0.036
No	19	13 (68.4%)	
HPV			
(+)	55	49 (89.1%)	0.022
(−)	5	2 (40.0%)	

^a^ClC3(+) denotes overexpression of ClC3 protein

^b^By Pearson’s χ^2^ test or Fisher’s exact test

^c^WDSCC, Highly differentiated squamous carcinoma; MDSCC, moderately differentiated squamous cell carcinoma; PDSCC, poorly differentiated squamous cell carcinoma

### ClC-3 is a prognostic survival biomarker for cervical carcinoma patients

**Fig. 3:**
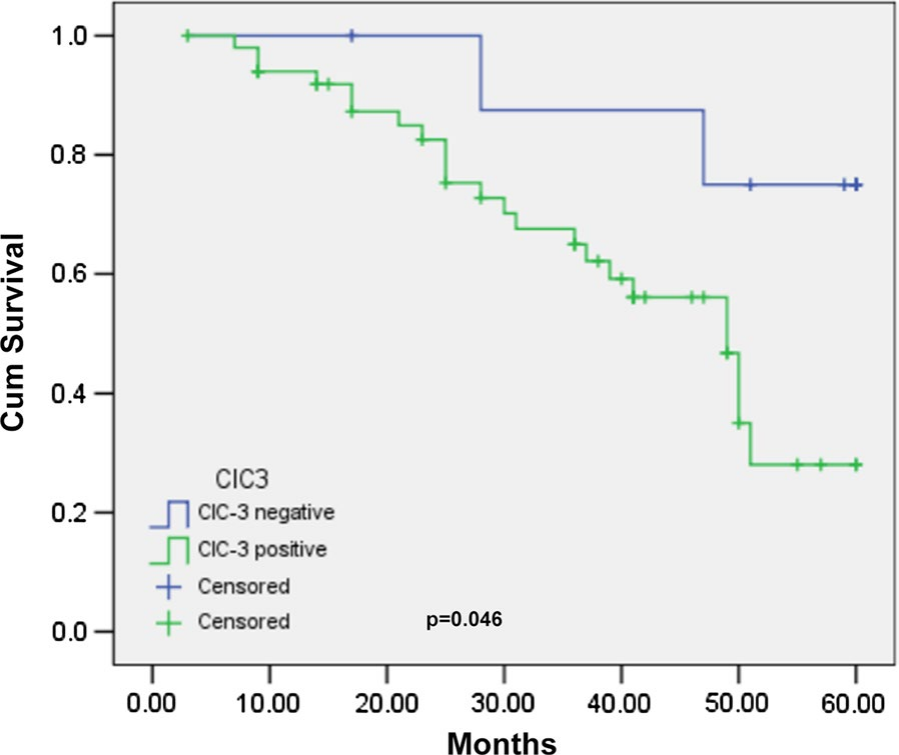
The relationship between ClC-3 expression and cumulative survival in cervical cancer patients. The Kaplan–Meier curve shows that expression of ClC-3 is oppositely correlated with the 5-year cumulative survival rate (Log-rank p = 0.046). 1: ClC-3 negative expression; 2: ClC-3 positive expression

To investigate whether ClC-3 expression influences long-term patient survival, we monitored 60 patients with squamous cervical carcinoma for 60 months and found the average survival time was 48.23 months. From the total, 19 patients died of carcinoma recurrence in the follow-up period (36.7%). The patient 5-year cumulative survival rate was 51.0%. As displayed in Fig. 3, compared with the ClC3 negative expression, the prognosis of cervical cancer was poorer in patients with ClC-3 positive tissues as demonstrated by the log-rank test (Log-rank p = 0.046). These data suggest that ClC-3 gene expression may be a prognostic biomarker for cervical carcinoma patients.

### ClC-3 expression in cervical cancer and normal cervical epithelial cell lines

**Fig. 4:**
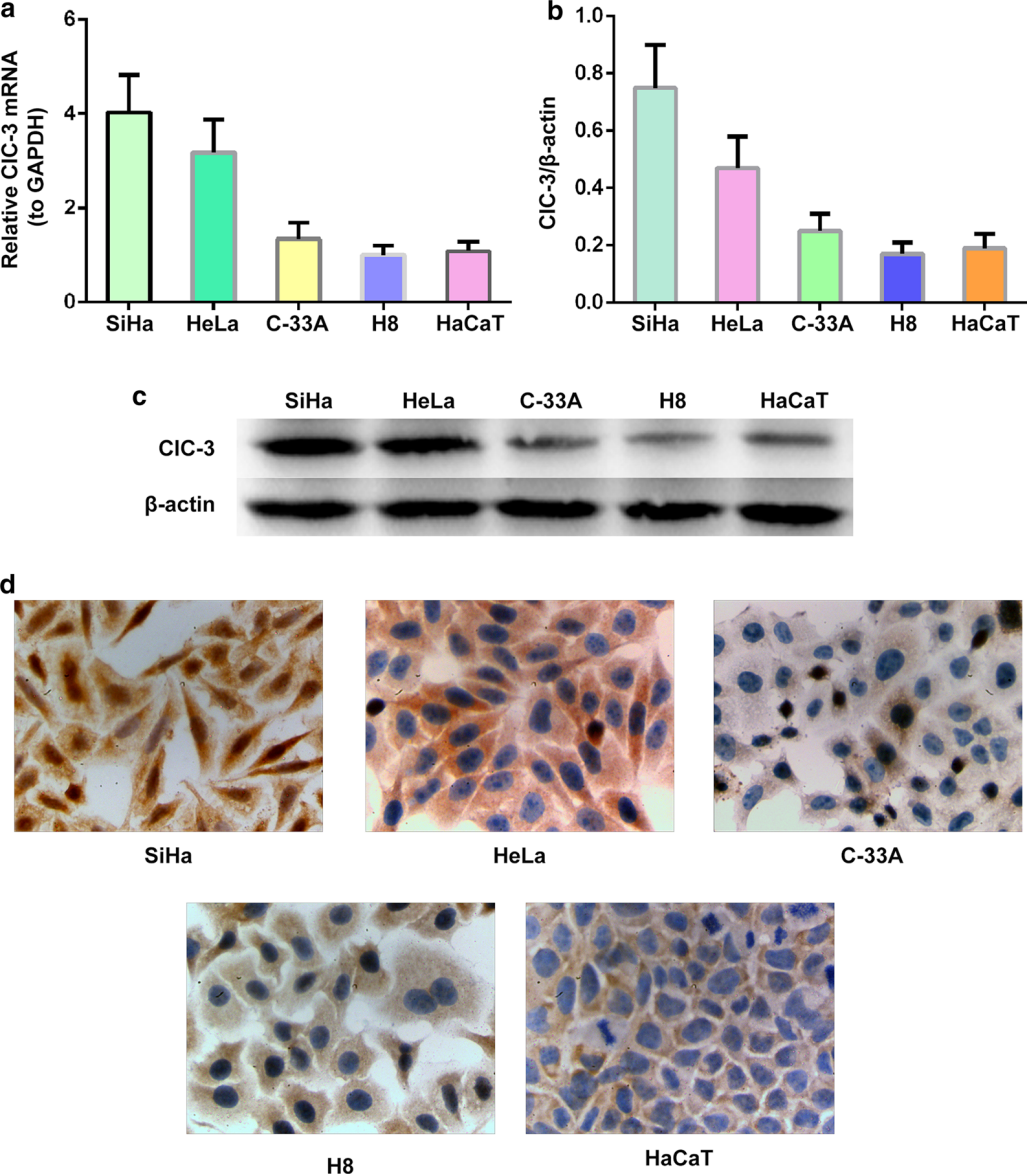
Increased expression of the ClC-3 mRNA and protein in SiHa and HeLa cell lines. **a** ClC-3 mRNA expression levels detected by quantitative real time RT-PCR in the SiHa, HeLa, C-33A, normal cervical epithelial cell line H8 and normal keratinocyte cell line HaCaT. **b** Densitometric analysis of the ClC-3 protein expression levels in SiHa, HeLa, C-33A, normal cervical epithelial cell line H8 and normal keratinocyte cell line HaCaT by western blotting. **c** The representative western blot of the ClC3 and β-actin proteins in the SiHa, HeLa, C-33A, H8 and HaCaT cell lines. The data in **a** and **b** are shown as the mean ± SD. **d** Images of the SiHa, HeLa, C-33A, normal cervical epithelial cell line H8 and normal keratinocyte cell line HaCaT, show the ClC-3 immunohistochemical staining, respectively

To further evaluate expression characteristics of ClC-3 gene, we tested ClC-3 protein and mRNA expression levels of three representative cervical carcinoma cell lines including SiHa, HeLa, C-33A, normal cervical epithelial cell line H8 and normal keratinocyte cell line HaCaT via western blotting and quantitative real-time PCR. As displayed in Fig. 4a, increasing expression levels of ClC-3 mRNA were detected in cell lines SiHa (4.03 ± 0.80, p < 0.01) and HeLa (3.18 ± 0.69, p < 0.01) but not in C-33A (1.35 ± 0.34, p > 0.05) compared with that of the H8 (1.01 ± 0.20) and HaCaT (1.08 ± 0.21) cell lines. However, there was no significant difference between the HeLa and Siha cell lines (p > 0.05).

Furthermore, we measured the ClC-3 protein level in HeLa, SiHa, C-33A, H8 and HaCaT cell lines by western blot and immunohistochemistry analysis (Fig. 4b, c). The data showed the expression levels of ClC-3 protein were consistent with the ClC-3 mRNA expression levels, suggesting that ClC-3 protein levels in HeLa and SiHa cells were also significantly higher in H8, HaCaT and C-33A cells; further, the ClC-3 protein stain was mainly observed in the cytoplasmic area. These data were consistent with in vivo data and suggested that ClC-3 is overexpressed in SiHa and HeLa but not C-33A human cancer cell lines, and H8 and HaCaT cell lines show low expression of ClC-3.

## Discussion

Although ClC-3 plays a well-known and major role in multiple cellular functions, the function of ClC-3 in the progression of cervical cancer has not been well studied. The present study investigated the relationship between the ClC-3 gene expression levels and its underlying clinical significance in human cervical carcinoma to illustrate its impact in the progression of cervical cancer.

ClC-3 has been confirmed to play an important role in cell volume, proliferation, migration and invasion of diversified tumour cells. In the present study, we found that: (i) Expression levels of ClC-3 mRNA and protein are significantly increased by 60% in paracancerous and cervical carcinoma tissues. The ClC-3 protein is expressed mainly in cervical epithelial cell cytoplasm, indicating ClC-3 is closely associated with cervical epithelial cell homeostasis and malignancy; (ii) We discovered the expression of ClC-3 was closely associated with patient clinical pathological characteristics, suggesting ClC-3 may be a potential biomarker for cervical carcinoma. Furthermore, overexpression of ClC-3 was significantly related to poor survival in cervical carcinoma, indicating it may be a latent predictor of cervical carcinoma prognosis; (iii) In vitro, ClC-3 was highly expressed in both SiHa and HeLa cell lines but it was expressed weakly in C-33A and H8 cell lines. Since C-33A derives from HPV negative cervical cancer, the carcinoma cells biological behaviour could change greatly. Taken together, we conjecture that ClC-3 may be associated with distant tumour metastasis.

Though there are many studies that investigate the roles of ClC-3 in the pathogenesis and pathophysiology of normal and cancer cells, the functions of ClC-3 in human cervical cancer progression are not well known. The ClC-3 gene is considered to be related to multiple tumours. The migration and invasion of tumour cells are primary processes in neoplasm metastasis [18], which requires changes in the tumour cell volume that demands ion channels to regulate ions and water through the plasma membrane [19]. Several studies showed that ClC-3 takes part in the regulation of cell volume changes and volume-regulated chloride currents which positively correlated with cell migration and invasion in cancer cells; this function disappeared after the knockout of ClC-3 [20–22]. In addition, glioma cells invade the normal brain by means of the activities of ClC-3 ion channels that promote cell volume dynamic regulation. ClC-3 is highly expressed on the human glioma cell plasma membrane, and its activity is regulated by phosphorylation via Ca(2+)/calmodulin-dependent protein kinase II (CaMKII) [23]. Downregulated ClC-3 expression by shRNA can decrease Ca^2+^-activated chloride current regulation through CaMKII and reduce bradykinin-induced human glioma cell migration. ClC-3 is located in the cytoplasm, nuclei, and plasma membrane of nasopharyngeal cancer (CNE-2Z) cells [24]; inhibition of ClC-3 protein expression was found to inhibit CNE-2Z cell proliferation and migration [25]. ClC-3 can move between the cytoplasm and cytoplasmic membrane; high expression of ClC-3 on the cell membrane may play a role in regulating cell volume [26] and facilitate tumour cell metastasis [27]. In our study, the localization of ClC-3 seems to differ depending on lymph node metastasis; ClC-3 is mainly expressed in the cytoplasm in lymph node cells without tumour metastasis, while ClC-3 is mainly expressed in the cytoplasmic membrane in lymph node cells with tumour metastasis (Fig. 2). We speculated that ClC-3 was involved in the cell volume regulation of enlarged lymph nodes with tumour metastasis. These data suggest that ClC-3 plays a crucial role in human tumour cell migration and invasion. In our present study, it has been demonstrated that ClC-3 may be closely associated with cervical cancer metastasis.

Our study also found that ClC-3 expression was related to cervical cancer HPV infection. The potential mechanism is still unclear. Interestingly, researches have shown that Cl− currents activated by volume-sensitive chloride channels may play a crucial role leading to regulatory volume decrease (RVD) in human cervical cancer [28], Ano1 is a Ca^2+^ activated Cl(−) channel that produces Ca^2+^ activated Cl(−) currents and is related to the human papilloma virus (HPV) infection and cancer [29]. This study suggests that Cl(−) currents may be involved in the process of HPV infection. ClC-3 is a member of the ClC superfamily of voltage-sensitive Cl− channels [30], a regulator of the volume-sensitive Cl− channel chloride currents [27] and may be responsible for HPV infection and cervical cancer development, although more research is needed to confirm this in the future.

Interestingly, we found a positive association between the expression of ClC-3 and the survival time. There are a few studies on ClC-3 and cancer survival time. Wang [9] found that ClC-3 was highly expressed in glioma tissues and was positively related to histological grade. Glioma patients with lower ClC-3 expression had a longer survival time, whereas patients with higher ClC-3 expression had a shorter survival time. ClC-3 expression is associated with invasion and metastasis, which may have a potential impact on survival [17]. Using ShClC-3 adenovirus, the ClC-3 expression was downregulated, which can significantly reduce glioma cell volume-regulated chloride currents, reduce MMP-9 and MMP-3 expression by inhibiting the transcriptional activity of Nuclear Factor-kappa B (NF-kappa B), and reduce the migration and invasion ability of glioma cells [9]. Xu [11] also reported that cytoplasmic ClC-3 overexpression is positively correlated with cervical cancer metastasis, and patients with high-grade ClC-3 expression in the cytoplasm showed poor survival. A possible mechanism involves ClC-3 overexpression in the cytoplasm of metastatic cancer cells and regulation of membrane ruffling by adjusting keratin 18 phosphorylation and controlling recirculation of beta1 integrin, which expedited cell migration and tumour metastasis. Furthermore, ClC-3 was located at different sites in different cell cycle and proliferative states. ClC-3 was mainly located in the cytoplasm during the late G2 DNA synthesis before active mitosis and hyperplasia [26]. In our study, we discovered that the localization of the chloride channel-3 is mainly in the cytoplasm of cervical cancer cells rather than the cytoplasmic membrane (Fig. 1F), and patients with overexpression of ClC-3 had significantly poor survival, indicating it may be an underlying predictor for cervical cancer prognosis.

## Conclusions

In conclusion, our results provide, for the first time, clinical and experimental evidence that ClC-3 expression is closely associated with cervical carcinoma development and poor survival, indicating ClC-3 may be a patent tumour marker, an expected prognostic indicator and a latent therapeutic target for cervical carcinoma.

## Abbreviations

ClC-3: chloride channel-3
IHC: immunohistochemistry assay
SCC: squamous cell carcinoma
LSIL: low-squamous intraepithelial lesions
HSIL: high-squamous intraepithelial lesions
HPVs: human papillomaviruses
MMP: matrix metalloproteinases
DAB: diaminobenzidine
DMEM: Dulbecco’s modified Eagle’s medium
ECL: enhanced chemiluminescence
HRP: horseradish peroxidase
IHC: immunohistochemistry
NC: negative control
PN: paracancerous normal cervical tissues
NF-κB: nuclear factor kappa-light-chain-enhancer of activated B cells
LP: lymph node metastasis positive
LN: lymph node metastasis negative
qRT-PCR: quantitative real-time PCR
SD: standard deviation
SDS-PAGE: sodium dodecyl sulfate polyacrylamide gel electrophoresis
